# Clinical Spectrum of LIG4 Deficiency Is Broadened with Severe Dysmaturity, Primordial Dwarfism, and Neurological Abnormalities

**DOI:** 10.1002/humu.22436

**Published:** 2013-09-18

**Authors:** Hanna IJspeert, Adilia Warris, Michiel Flier, Ismail Reisli, Sevgi Keles, Sandra Chishimba, Jacques JM Dongen, Dik C Gent, Mirjam Burg

**Affiliations:** 1 Department of Immunology, Erasmus MC, University Medical Center RotterdamRotterdam, The Netherlands; 2 Department of Pediatrics, Erasmus MCRotterdam, The Netherlands; 3 Department of Pediatrics and the Nijmegen Institute for Infection, Inflammation and Immunity, Radboud University Nijmegen Medical CenterNijmegen, The Netherlands; 4 Department of Pediatric Immunology and Allergy, Necmettin Erbakan University Meram Medical FacultyKonya, Turkey; 5 Department of Genetics, Erasmus MC, University Medical Center RotterdamRotterdam, The Netherlands

**Keywords:** LIG4, immunodeficiency, primordial dwarfism, non-homologous end joining, NHEJ

## Abstract

DNA double-strand break repair via non-homologous end joining (NHEJ) is involved in recombination of immunoglobulin and T-cell receptor genes. Mutations in NHEJ components result in syndromes that are characterized by microcephaly and immunodeficiency. We present a patient with lymphopenia, extreme radiosensitivity, severe dysmaturity, corpus callosum agenesis, polysyndactily, dysmorphic appearance, and erythema, which are suggestive of a new type of NHEJ deficiency. We identified two heterozygous mutations in *LIG4*. The p.S205LfsX29 mutation results in lack of the nuclear localization signal and appears to be a null mutation. The second mutation p.K635RfsX10 lacks the C-terminal region responsible for XRCC4 binding and LIG4 stability and activity, and therefore this mutant might be a null mutation as well or have very low residual activity. This is remarkable since *Lig4* knockout mice are embryonic lethal and so far in humans no complete LIG4 deficiencies have been described. This case broadens the clinical spectrum of LIG4 deficiencies.

The non-homologous end joining (NHEJ) pathway is involved in the repair of the DNA double-strand breaks. These can be generated during DNA replication, exposure to exogenous agents such as ionizing radiation (IR), or physiologically during V(D)J recombination, as happens during the early stages of B- and T-cell differentiation to generate antigen-specific B- and T-cell receptors. Defects in NHEJ factors result in IR sensitivity, and in defects in V(D)J recombination leading to immunodeficiency. Genetic defects have been described in several NHEJ genes, including *DCLRE1C* (MIM #605988), *PRKDC* (MIM #600899), *NHEJ1* (MIM #611290), and *LIG4* (MIM #601837) [Moshous et al., 2001; Noordzij et al., 2003; O'Driscoll et al., 2004; van der Burg et al., 2009; van der Burg et al., 2006]. To date, 16 LIG4 deficient patients have been described [Ben-Omran et al., 2005; Buck et al., 2006; Enders et al., 2006; Grunebaum et al., 2008; O'Driscoll et al., 2001; Riballo et al., 1999; Toita et al., 2007; Unal et al., 2009; van der Burg et al., 2006; Yue et al., 2013] (summarized in Supp. Table S1). All patients were IR sensitive, but clinically they can be divided into five distinct disease categories: (1) leukemia, (2) LIG4 syndrome (MIM #606593), (3) Dubowitz syndrome (MIM #223370), (4) Omenn syndrome (MIM #603554), and (5) radiosensitive severe combined immunodeficiency (MIM #602450). Here, we present a male patient with a new clinical phenotype of LIG4 deficiency characterized by microcephalic primordial dwarfism and neurological abnormalities.

The patient was born with extreme dysmaturity after 37 weeks of gestational age. At the age of 3 months, his height was 43 cm (−7.4 SD), weight was 1870 g (−8.9 SD), and head circumference was 29 cm (−8.9 SD). Besides the dysmaturity, the patient had several dysmorphisms (Fig. 1A and B) including hypotelorism, small viscerocranium, flat philtrum, thin upper lip, preaxial polydactyly (duplication of distal phalanx of left thumb), brachymesophalangy of the digits V on both hands, and partial cutaneous syndactyly of digits II–V of both feet (Fig. 1C and D), dysplastic kidneys with bilaterally vesicourethral reflux and urethral valves. Additionally, the patient had the neurological abnormalities, corpus callosum dysgenesia, and colpocephaly. At the age of 2 and 4 months, he suffered from a *Pseudomonas aeruginosa* and *Enterococcus faecalis* urinary tract infection, respectively, and he tested positive for *P. aeruginosa*, *P. jiroveci*, rhinovirus, norovirus, astrovirus, *Clostridium difficile*, and *Candida*. Besides the infectious complications, the first 3 months of life were characterized by feeding difficulties, diarrhea, failure to thrive, cholestatic icterus, tubulopathy, generalized erythema, and very dry cracked skin. Initially the patient seemed to recover from the opportunistic infections, but a second episode of an acute sepsis-like syndrome with respiratory insufficiency complicated by severe gastrointestinal bleeding—probably due to the development of thrombocytopenia—could not be successfully treated; the patient died at the age of 6 months.

**Figure 1:**
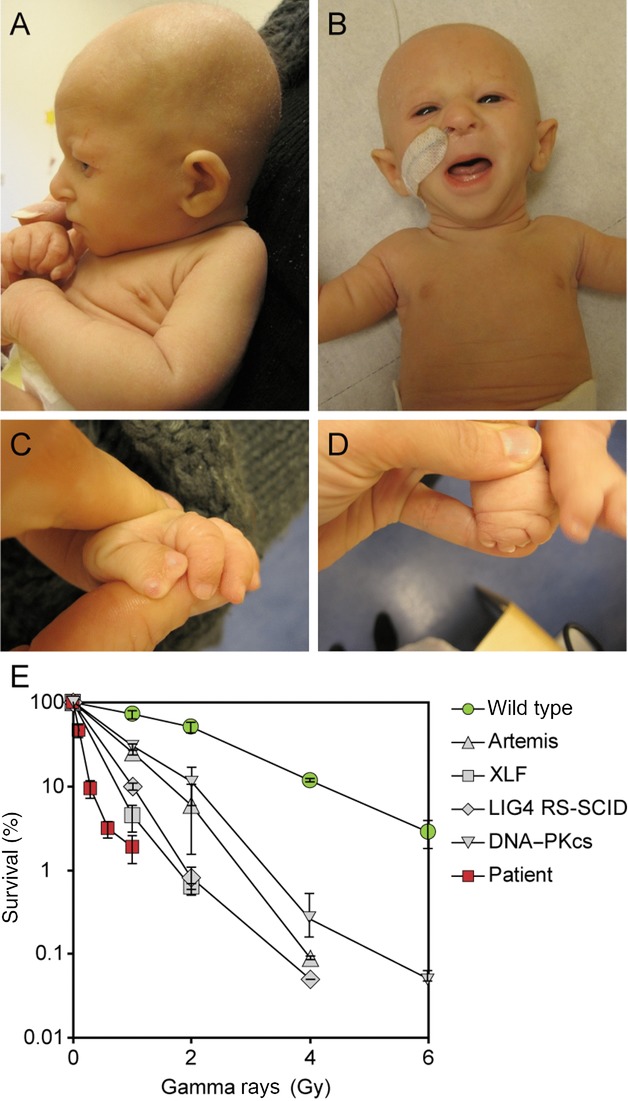
Dysmorphic features of the face, hand, and feet and ionizing radiation sensitivity. The patient presented with facial dysmporphisms including beaked nose (A), hypotelorism, small viscerocranium, flat philtrum, and thin upper lip (B). In addition, the patient had a duplication of distal phalanx of left thumb, brachymesophalangy of the digits V on both hands (C), and partial cutaneous syndactyly of digits II–V of both feet (D). Clonogenic survival assay of wild-type (C5RO) fibroblasts and patients’ fibroblasts deficient for Artemis, DNA–PKcs, XLF, or LIG4 (LIG4 SCID). The patient was extremely sensitive for ionizing radiation. Each curve represents the mean of at least two independent experiments. Error bars represent SEM (E).

Immunologic evaluation showed normal numbers of NK cells, very low B-cell numbers, and increased T-cell numbers (Supp. Table S2 and Supp. Materials and Methods). The increase in the number of T cells was mainly caused by an increase in the CD8+ T cells probably related to a viral infection. The presence of maternal T cells was excluded. Immunoglobulin (Ig) G was decreased, which was not secondary to malabsorption, whereas IgM and IgA were normal (Supp. Table S2) and Ig substitution therapy was initiated at the age of 4.5 months.

The clinical presentation, especially the immunodeficiency together with microcephaly was suggestive for a NHEJ defect. Therefore, the patient's fibroblasts were tested in a clonogenic survival assay (Supp. Materials and Methods). These were extremely IR sensitive by an order of magnitude, c.f. the control at 10% survival (Fig. 1E) and even more sensitive than those of LIG4 and XLF deficient patients (three times more sensitive than the control at 10% survival), which are normally more IR sensitive than fibroblasts from Artemis and DNA–PKcs deficient patients (Fig. 1E). This result was indicative for a severe NHEJ defect.

Sequencing of the *LIG4* gene (Supp. Materials and Methods) showed the presence of two heterozygous single-nucleotide deletions in the *LIG4* gene (c.613delT and c.1904delA) (submitted to http://www.lovd.nl/LIG4). The first deletion was inherited from the mother and resulted in a frameshift and a premature stop codon in the DNA-binding domain (p.S205LfsX29). This mutation was recently described in the LIG4 patient presenting with the Dubowitz syndrome [Yue et al., 2013]. The mutant LIG4 protein lacks the nuclear localization signal (NLS), the active site, the adenylation domain, the oligo-binding domain, both BRCT motifs and the XRCC4-binding site (Fig. 2A). Since LIG4 exerts its function in the nucleus, we investigated the localization of the mutant LIG4 proteins by using green fluorescent protein (GFP)-tagged LIG4 expression constructs (Fig. 2A and Supp. Material and Methods). In contrast to wild-type LIG4, the S205LfsX29 LIG4 mutant was only expressed in the cytoplasm (Fig. 2B), which indicates that the S205LfsX29 mutant represents a null mutation.

**Figure 2:**
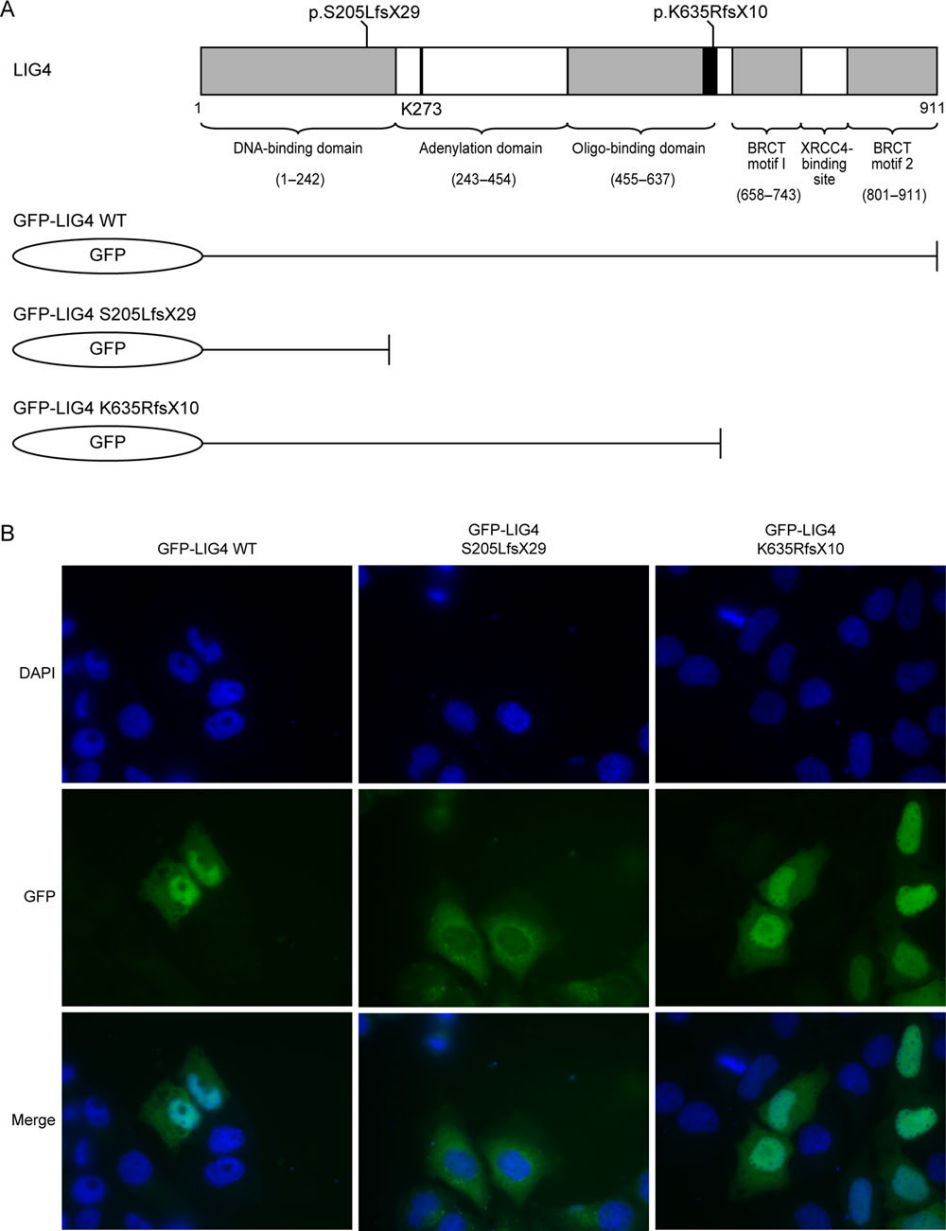
LIG4 mutants and their expression. Schematic representation of the LIG4 protein (NM_001098268.1) and the GFP-LIG4 expression constructs. The different domains, active site (K273), and mutations identified in the patient are indicated. The nuclear localization signal (NLS1 [P_623_QEKKRK_629_] and NLS2 [A_630_APKMKKVI_638_] [Girard, et al., 2004]) is indicated in black. The numbers between brackets indicated the amino acid position (A). Localization of GFP-LIG4 wild type and mutants after transient transfection of U2OS cells (B).

The second paternally inherited deletion resulted in a frameshift, changing the last four amino acids of the NLS (K_635_K_636_V_637_I_638_ → R_635_K_636_L_637_L_638_) without affecting the charge, and a premature stop codon (p.K635RfsX10). In this mutant, part of the NLS is retained, but it lacks both BRCT motifs and the XRCC4-binding site, which are necessary for the interaction with Cernunnos/XLF [Critchlow et al., 1997]. LIG4 interacts with XRCC4 and forms a 1:2 complex [Sibanda et al., 2001]. The interaction with XRCC4 is important since it stabilizes LIG4 protecting it from degradation [Bryans et al., 1999]. This implies that the p.K635RfsX10 mutant has probably very low residual activity or might even be a null mutant.

In our overexpression system, this mutant was still expressed in the nucleus (Fig. 2B) and is therefore consistent with the results of Girard et al. (2004) who found that deleting both BRCT motifs and the XRCC4-binding domain (Δ653–911) still resulted in nuclear expression of the mutant LIG4 protein (Girard et al., 2004). None of the reported *LIG4* mutations in patients retains the NLS but lacks the XRCC4 interaction domain (Supp. Table S1 and Supp. Fig. S1). The p.R814X mutant lacks the BRCT 2 motif, but the NLS and XRCC4-binding site are present [Ben-Omran et al., 2005; O'Driscoll, et al., 2001]. This mutant is expressed in the nucleus and retained ∼10%–15% residual double-strand ligation activity, but was barely detectable in the patient [O'Driscoll et al., 2001]. The estimated residual activity of this mutant is <1% [Girard et al., 2004]. The p.R580X mutant lacks the NLS and the XRCC4 interaction domain. Since this mutant is not stably expressed, does not interact with XRCC4, and does not enter the nucleus, it is considered to be a null mutant. Similar to the p.R580X mutant, the p.K635RfsX10 mutant lacks XRCC4-interacting domain [Critchlow et al., 1997], which is necessary for LIG4 stability and protection of LIG4 from degradation [Bryans, et al., 1999]. Based on these data and the severity of the clinical phenotype of the patient, we expect that this mutant has even less residual activity than the LIG4 mutants described before and might represent a null mutation. This is remarkable since LIG4 is considered to be essential for humans and Lig4 knockout mice are embryonic lethal [Barnes et al., 1998; Frank et al., 1998]. This study shows that LIG4 mutations affect the immune system or neurological development with different severity.
