# Supplementary material for: Clinical Spectrum of LIG4 Deficiency Is Broadened with Severe Dysmaturity, Primordial Dwarfism, and Neurological Abnormalities
Source: Hum Mutat. 2013 Sep 18;34(12):1611–4. doi: 10.1002/humu.22436 (PMC3910166; doi:10.1002/humu.22436)
Supplement: Supplementary file 1 [file humu0034-1611-sd1.pdf]

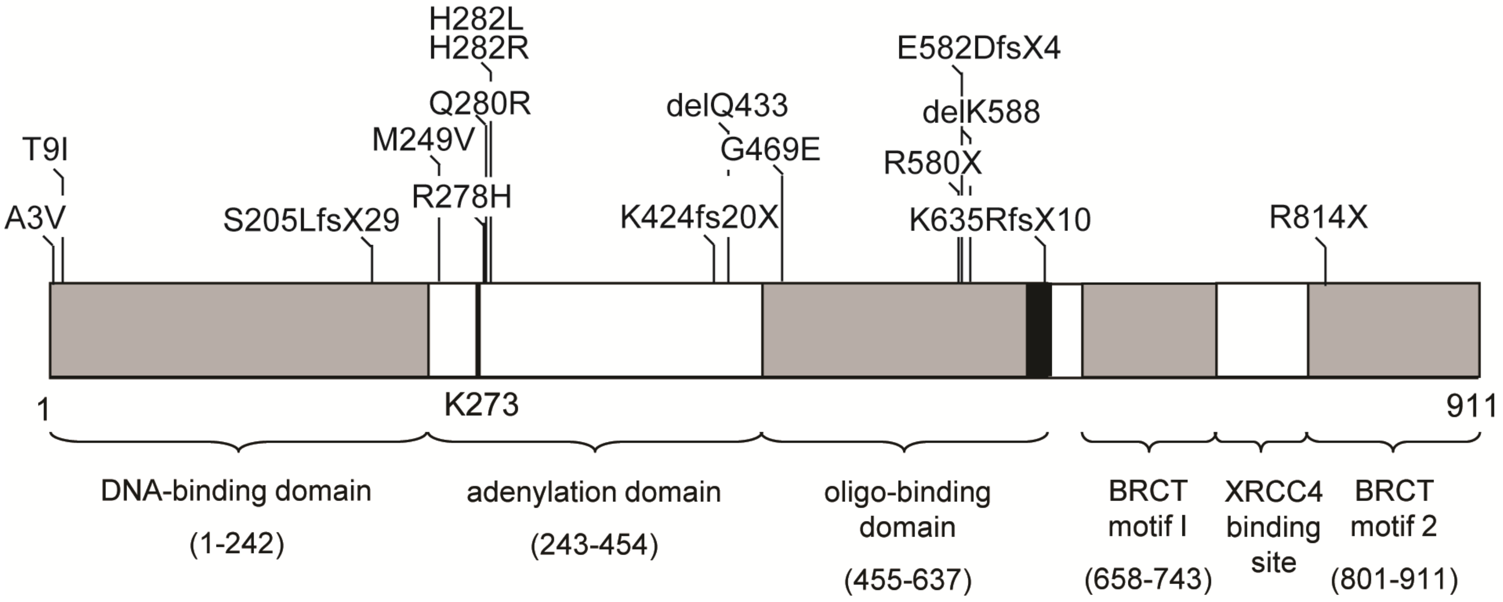

Supp. Figure S1. Localization of *LIG4* mutations. Schematic representation of all LIG4 mutations described in literature, the patient described here and our unpublished patient (see Supp. Table S1).

Supp. Table S1. Overview of LIG4 deficient patients (new and literature)

	Age at diagnosis	Mutations		Radiosensitivity	Malignancy	Microcephaly	Cytopenia	Immunodeficiency	Developmental delay	Reference
**Leukemia**										
180BR	14y	p.R278H	homoz.	+	leukemia	?	?	-	-	(Riballo, et al., 1999)
**LIG4 syndrome**										
3703	4.5y	p.R814X	homoz.	++	leukemia	+	+	-	+	(Ben-Omran, et al., 2005)
Patient	14y	p.M249V, p.A3V,p.T9I	p.K424fs20X	++	leukemia	+	+	+	+	(Toita, et al., 2007)
411BR	9y	p.R278H	homoz.	+	-	(+)	+	+	+	(O'Driscoll, et al., 2001)
2303	46y	p.R580X	p.R814X	+	-	+	+	+	?	(O'Driscoll, et al., 2001)
2304	48y	p.R580X	p.R814X	+	-	+	?	+	?	(O'Driscoll, et al., 2001)
99P0149	9y	p.G469E	p.R814X	+	-	+	+	+	+	(O'Driscoll, et al., 2001)
Case 1	4y	p.delK588	homoz.	?	-	+	+	+	+	(Unal, et al., 2009)
Case 2	3.5y	p.delK588	homoz.	?	-	+	+	+	+	(Unal, et al., 2009)
LIG4-4	4 months	p.H282R	homoz.	?	-	+	+	+	+	new
**Dubowitz syndrome**										
Patient	1y	S205LfsX29	p.R814X	++	carcinoma	+	?	?	?	(Yue, et al., 2013)
**Omenn's syndrome**										
Patient	3wk	p.H282L	p.E582DfsX4	?	-	+	?	+	?	(Grunebaum, et al., 2008)
**RS-SCID**										
P1	2y	p.H282L	p.K424fs20X	+	+	+	+	+++	-	(Enders, et al., 2006)
P2	4 wk	p.H282L	p.K424fs20X	+	-	+	-	+++	+	(Enders, et al., 2006)
P-1	1.5y	p.Q280R	p.K424fs20X	+	-	+	-	+++	-	(Buck, et al., 2006)
P-2	4 wk	p.Q280R	p.K424fs20X	+	-	+	-	+++	?	(Buck, et al., 2006)
LIG4-1	1.5y	p.delQ433	homoz.	+	-	-	+	+++	-	(van der Burg, et al., 2006)
**Patient**	17wk	S205LfsX29	K635RfsX10	++	-	+	+	+++	+	new

Supp. Table S2. Immunophenotyping of the patient

	**6 months ($x10^9$/L)**	**Reference values ($x10^9$/L) (Comans-Bitter, et al., 1997)**
**CD3**	5.73	2.4-6.9
**CD4**	3.38	1.5-5.0
**CD4CD45RA/RO**	0.10/3.31	RA>RO
**CD8**	2.41	0.5-1.6
**CD8CD45RA/RO**	0.10/2.3	RA>RO
**CD19**	0.06	0.6-3.0
**CD56**	0.31	0.1-1.3
	**4 months (g/L)**	**Reference values 5-6 months**
**IgG**	0.76	2.6-15.2
**IgM**	0.22	0.07-0.65
**IgA**	0.85	0.08-0.90

Supp. Materials and Methods

Cell samples and flow cytometric immunophenotyping

Peripheral blood and a skin biopsy were obtained with informed consent and according to the guidelines of the local Medical Ethics Committees. Flow cytometric analysis of peripheral blood was performed as described previously (Noordzij, et al., 2003; van der Burg, et al., 2006).

Cell lines and tissue culture

Primary fibroblasts were cultured from a skin biopsy from the patient in addition to fibroblasts from a healthy control (C5RO), Artemis-deficient (Artemis-6) (van der Burg, et al., 2007), XLF deficient (XLF-5) and DNA-PKcs deficient patients (van der Burg, et al., 2009) and a LIG4 SCID patient (van der Burg, et al., 2006). Fibroblasts were cultured in DMEM (BioWhittaker, Walkersville, MD, USA), supplemented with 10% FCS, penicillin (100 U/ml), and streptomycin (100 μg/ml).

Clonogenic survival assay and sequence analysis

Clonogenic survival assay was performed as described previously (van der Burg, et al., 2009). *XLF(NHEJ1)* (NM_024782.2) and *LIG4* (NM_001098268.1) were amplified by PCR and sequenced according to (van der Burg, et al., 2009).

Localization of LIG4 mutants

Mutant GFP-LIG4 expression constructs (S205LfsX29 and K635RfsX9) were made by using the QuickChange site directed mutagenesis kit (Agilent Technologies, Santa Clara, CA, USA) using the GFP-LIG4 WT as the parent plasmid. U2OS cells were transfected using Fugene 6 (Promega, Madison, WI, USA). After 24h cells were fixed with 2% paraformaldehyde and expression was determined using a fluorescence microscope.

Supp. References

Ben-Omran TI, Cerosaletti K, Concannon P, Weitzman S, Nezarati MM. 2005. A patient with mutations in DNA Ligase IV: clinical features and overlap with Nijmegen breakage syndrome. Am J Med Genet A 137A:283-7.

Buck D, Moshous D, de Chasseval R, Ma Y, le Deist F, Cavazzana-Calvo M, Fischer A, Casanova JL, Lieber MR, de Villartay JP. 2006. Severe combined immunodeficiency and microcephaly in siblings with hypomorphic mutations in DNA ligase IV. Eur J Immunol 36:224-35.

Comans-Bitter WM, de Groot R, van den Beemd R, Neijens HJ, Hop WC, Groeneveld K, Hooijkaas H, van Dongen JJ. 1997. Immunophenotyping of blood lymphocytes in childhood. Reference values for lymphocyte subpopulations. J Pediatr 130:388-93.

Enders A, Fisch P, Schwarz K, Duffner U, Pannicke U, Nikolopoulos E, Peters A, Orlowska-Volk M, Schindler D, Friedrich W, Selle B, Niemeyer C*et al*. 2006. A severe form of human combined immunodeficiency due to mutations in DNA ligase IV. J Immunol 176:5060-8.

Grunebaum E, Bates A, Roifman CM. 2008. Omenn syndrome is associated with mutations in DNA ligase IV. J Allergy Clin Immunol 122:1219-20.

Noordzij JG, Verkaik NS, van der Burg M, van Veelen LR, de Bruin-Versteeg S, Wiegant W, Vossen JM, Weemaes CM, de Groot R, Zdzienicka MZ, van Gent DC, van Dongen JJ. 2003. Radiosensitive SCID patients with Artemis gene mutations show a complete B-cell differentiation arrest at the pre-B-cell receptor checkpoint in bone marrow. Blood 101:1446-52.

O'Driscoll M, Cerosaletti KM, Girard PM, Dai Y, Stumm M, Kysela B, Hirsch B, Gennery A, Palmer SE, Seidel J, Gatti RA, Varon R*et al*. 2001. DNA ligase IV mutations identified in patients exhibiting developmental delay and immunodeficiency. Mol Cell 8:1175-85.

Riballo E, Critchlow SE, Teo SH, Doherty AJ, Priestley A, Broughton B, Kysela B, Beamish H, Plowman N, Arlett CF, Lehmann AR, Jackson SP*et al*. 1999. Identification of a defect in DNA ligase IV in a radiosensitive leukaemia patient. Curr Biol 9:699-702.

Toita N, Hatano N, Ono S, Yamada M, Kobayashi R, Kobayashi I, Kawamura N, Okano M, Satoh A, Nakagawa A, Ohshima K, Shindoh M*et al*. 2007. Epstein-Barr virus-associated

B-cell lymphoma in a patient with DNA ligase IV (LIG4) syndrome. Am J Med Genet A 143:742-5.

Unal S, Cerosaletti K, Uckan-Cetinkaya D, Cetin M, Gumruk F. 2009. A novel mutation in a family with DNA ligase IV deficiency syndrome. Pediatr Blood Cancer 53:482-4.

van der Burg M, Ijspeert H, Verkaik NS, Turul T, Wiegant WW, Morotomi-Yano K, Mari PO, Tezcan I, Chen DJ, Zdzienicka MZ, van Dongen JJ, van Gent DC. 2009. A DNA-PKcs mutation in a radiosensitive T-B- SCID patient inhibits Artemis activation and nonhomologous end-joining. J Clin Invest 119:91-8.

van der Burg M, van Veelen LR, Verkaik NS, Wiegant WW, Hartwig NG, Barendregt BH, Brugmans L, Raams A, Jaspers NG, Zdzienicka MZ, van Dongen JJ, van Gent DC. 2006. A new type of radiosensitive T-B-NK+ severe combined immunodeficiency caused by a LIG4 mutation. J Clin Invest 116:137-45.

van der Burg M, Verkaik NS, den Dekker AT, Barendregt BH, Pico-Knijnenburg I, Tezcan I, vanDongen JJ, van Gent DC. 2007. Defective Artemis nuclease is characterized by coding joints with microhomology in long palindromic-nucleotide stretches. Eur J Immunol 37:3522-8.

Yue J, Lu H, Lan S, Liu J, Stein MN, Haffty BG, Shen Z. 2013. Identification of the DNA repair defects in a case of Dubowitz syndrome. PLoS One 8:e54389.
